# The efficacy and safety of ramucirumab plus docetaxel in older patients with advanced non‐small cell lung cancer

**DOI:** 10.1111/1759-7714.13429

**Published:** 2020-04-14

**Authors:** Tadashi Sakaguchi, Naoki Furuya, Kentaro Ito, Naoya Hida, Kei Morikawa, Yuko Komase, Takeo Inoue, Osamu Hataji, Masamichi Mineshita

**Affiliations:** ^1^ Division of Respiratory Medicine, Department of Internal Medicine St. Marianna University School of Medicine Kawasaki Japan; ^2^ Respiratory Center, Matsusaka Municipal Hospital Matsusaka Japan; ^3^ Division of Respiratory Medicine St. Marianna University School of Medicine Yokohama Japan

**Keywords:** Docetaxel, non‐small cell lung cancer, older patients, PEG‐G‐CSF, ramucirumab

## Abstract

**Background:**

Ramucirumab plus docetaxel (RAM+DOC) is expected to prolong survival in patients with advanced non‐small cell lung cancer (NSCLC); however, the efficacy and safety for older patients remains unknown. The objective of this study was to evaluate the efficacy and safety of RAM+DOC in patients 75 years and older.

**Methods:**

We retrospectively reviewed consecutive patients with advanced NSCLC who had received RAM+DOC treatment at three institutions. We compared the efficacy and safety in patients 75 years and older to those under 75 years of age.

**Results:**

A total of 114 patients were identified. The median progression‐free survival, time to treatment failure and overall survival was 3.6 (95% CI: 0.4–6.7), 3.1 (95% CI: 2.4–3.9) and 11.2 months (95% CI: 5.6–16.8) in the older group (*N* = 23), and 4.2 (95% CI: 3.3–5.0), 3.4 (95% CI: 3.3–5.0) and 12.2 months (95% CI: 9.1–15.4) in the younger group (*N* = 91), respectively. Survival curves were similar for each group, while the objective response rate was 30.4% (95% CI: 13.2–52.9%) in older patients and 35.2% (95% CI, 25.4–45.9%) for the younger group. A total of 22 older patients (95.7%) and 73 (80.2%) younger patients received primary prophylactic pegylated‐granulocyte‐colony stimulating factor (PEG‐G‐CSF). Four older patients (17.3%) and 14 younger patients (15.3%) discontinued RAM+DOC due to adverse events.

**Conclusions:**

RAM+DOC is expected to be efficacious and tolerable in older patients when supported with prophylactic PEG‐G‐CSF therapy.

**Key points:**

**Significant findings of the study**

・PFS, OS, and ORR in older patients were similar to those under 75 years of age.

・Safety of RAM+DOC was well tolerated in older patients with prophylactic PEG‐G‐CSF.

・Prophylactic PEG‐G‐CSF with RAM+DOC may contribute to better efficacy.

What this study adds

・This study suggests that RAM+DOC with prophylactic PEG‐G‐CSF is expected to be a useful option in older patients with advanced NSCLC.

## Introduction

As the world's population ages, older patients with non‐small‐cell lung cancer (NSCLC) are increasing in clinical practice. Chemotherapy is the standard treatment for patients with advanced and metastatic NSCLC. Targeted therapies for NSCLC patients harboring driver oncogene alterations are generally recommended regardless of age; therefore, cytotoxic agents are still indicated in those with driver oncogene alterations after targeted therapy. In a recent pooled analysis of older patients with advanced NSCLC with programmed death‐ligand 1 (PD‐L1) positive tumors, immune checkpoint inhibitors (ICI) improved overall survival (OS) compared with cytotoxic chemotherapy, with a more favorable safety profile.^1^ However, whether older patients derive similar benefits or can tolerate ICIs in clinical practice is uncertain. Although recent studies have shown efficacy for carboplatin‐based platinum doublets in older patients,^2^, ^3^, ^4^, ^5^ docetaxel monotherapy is a standard of care for chemotherapy‐naïve older patients with advanced NSCLC.^6^

Ramucirumab is a fully human immunoglobulin G1 monoclonal antibody that specifically binds to the vascular endothelial growth factor (VEGF) receptor‐2 extracellular domain with high affinity, preventing binding of all VEGF ligands and receptor activation.^7^ In the REVEL trial, ramucirumab plus docetaxel (RAM+DOC) improved objective response rates (ORR), progression‐free survival (PFS) and OS compared with docetaxel monotherapy as a second‐line treatment in patients with advanced NSCLC.^8^ Moreover, in the JVCG trial, a similarly designed Japanese randomized phase II trial, second‐line RAM+DOC improved PFS to that seen in the REVEL trial with a manageable safety profile.^9^

RAM+DOC is expected to prolong survival in patients with advanced NSCLC; however, the efficacy and safety in older patients is still unknown. Therefore, in this study, we retrospectively evaluated the efficacy and safety of RAM+DOC in patients 75 years and older.

## Methods

### Patient selection

This retrospective study was conducted across three institutions in Japan (St. Marianna University School of Medicine, Yokohama City Seibu Hospital and Matsusaka Municipal Hospital). We reviewed electronic data from consecutive patients with advanced NSCLC who received RAM+DOC from June 2016 to December 2018. Clinical data assessments included: patient characteristics; histology; clinical stage (UICC eighth edition); Eastern Cooperative Oncology Group performance status (ECOG PS); the number of prior treatments; prior bevacizumab or ICI therapies; usage of prophylactic pegylated‐granulocyte‐colony stimulating factor (PEG‐G‐CSF); treatment outcomes and adverse events. This study was approved by the institutional review board of each institution.

### Definition of older patients

We defined older patients as those 75 years and older according to guidelines from the Japan Lung Cancer Society.^10^ Although many studies and subgroup analyses in Western countries have assessed older patients as 70 years and older, we regard patients aged 70 to 75 years as treatable with platinum‐based chemotherapy in Japan.

### Drug administration

Intravenous ramucirumab (10 mg/kg) plus docetaxel (60 mg/m^2^) were administered every three weeks until disease progression or unacceptable toxicity. The use of primary and secondary prophylactic PEG‐G‐CSF and dose modification were at the discretion of the attending physicians.

### Treatment assessment

The data cutoff was April 2019. Efficacy endpoints were PFS, time to treatment failure (TTF), OS, ORR, and the disease control rate (DCR). Endpoints for safety included the incidence of grade ≥ 3 neutropenia and febrile neutropenia, adverse events leading to the discontinuation of treatment, and the incidence of treatment related death. The Response Evaluation Criteria in Solid Tumors, Version1.1 was used to assess the response to treatment.^11^ Adverse events were graded according to the National Cancer Institute Common Terminology Criteria for Adverse Events, version 4.03.

### Statistical analysis

PFS, TTF, and OS survival curves using the Kaplan‐Meier method compared the two groups by log‐rank test (older vs. younger patients). To identify prognostic factors, univariate and multivariate analyses were conducted. Of the selected factors, *P*‐values less than 0.1 in univariate analysis were included in multivariate analysis. Statistical analyses were performed using Student's *t*‐test and χ2 test, and Fisher's exact test for continuous and categorical variables. Statistical analyses were performed using SPSS software, version 23.0 (SPSS Inc., Chicago, USA). A *P*‐value less than 0.05 was considered statistically significant.

## Results

### Patient characteristics

A total of 114 patients (older, 23; younger, 91) were identified for efficacy and safety analyses. The main characteristics for each group are shown in Table 1, and a more detailed comparison of characteristics are displayed in Tables S1A and B. Among the older group, 47.8% were female, 52.2% were never smokers and 39.1% were diagnosed with squamous cell carcinoma. The use of RAM+DOC for early‐line (prior treatments = 0–1) was most prevalent in the older group (39.1%), while the use of late‐line (prior treatments = ≥3) was higher for the younger group (42.9%). In the older group, over half of the patients (56.5%) were administered RAM+DOC after ICI treatment.

**Table 1:** Patient characteristics

	Older group (≥75)	Younger group (<75)	
	(*N* = 23)	(*N* = 91)	*P*‐value
Median age (range)	77 (75–86)	68 (40–74)	<0.001*
Sex			
Male	12 (52.2%)	65 (71.4%)	0.087
Female	11 (47.8%)	26 (28.6%)	
Smoking history			
Never	12 (52.2%)	19 (20.9%)	0.007*
Former/current	11 (47.8%)	72 (79.1%)	
Histology			
Nonsquamous cell	14 (60.9%)	73 (80.2%)	0.06
Squamous cell	9 (39.1%)	18 (19.8%)	
ECOG PS			
0/1	22 (95.7%)	81 (89.0%)	0.458
2	1 (4.3%)	10 (11.0%)	
Clinical stage			
IIIA‐IIIC	2 (8.7%)	7 (7.7%)	0.929
IVA‐IVB	16 (69.6%)	66 (72.5%)	
Recurrence	5 (21.7%)	18 (19.8%)	
No.of prior treatments			
0–1	9 (39.1%)	18 (19.8%)	0.13
2	8 (34.8%)	34 (37.4%)	
≥3	6 (26.1%)	39 (42.9%)	
Prior bevacizumab			
Administered	13 (56.5%)	39 (42.9%)	0.253
None	10 (43.5%)	52 (57.1%)	
Prior ICI treatment			
Administered	13 (56.5%)	35 (38.5%)	0.156
None	10 (43.5%)	56 (61.5%)	

ECOG PS, Eastern Cooperative Oncology Group performance status; ICI, immune checkpoint inhibitor.

* *P* < 0.05.

### Efficacy analysis

**Figure 1:**
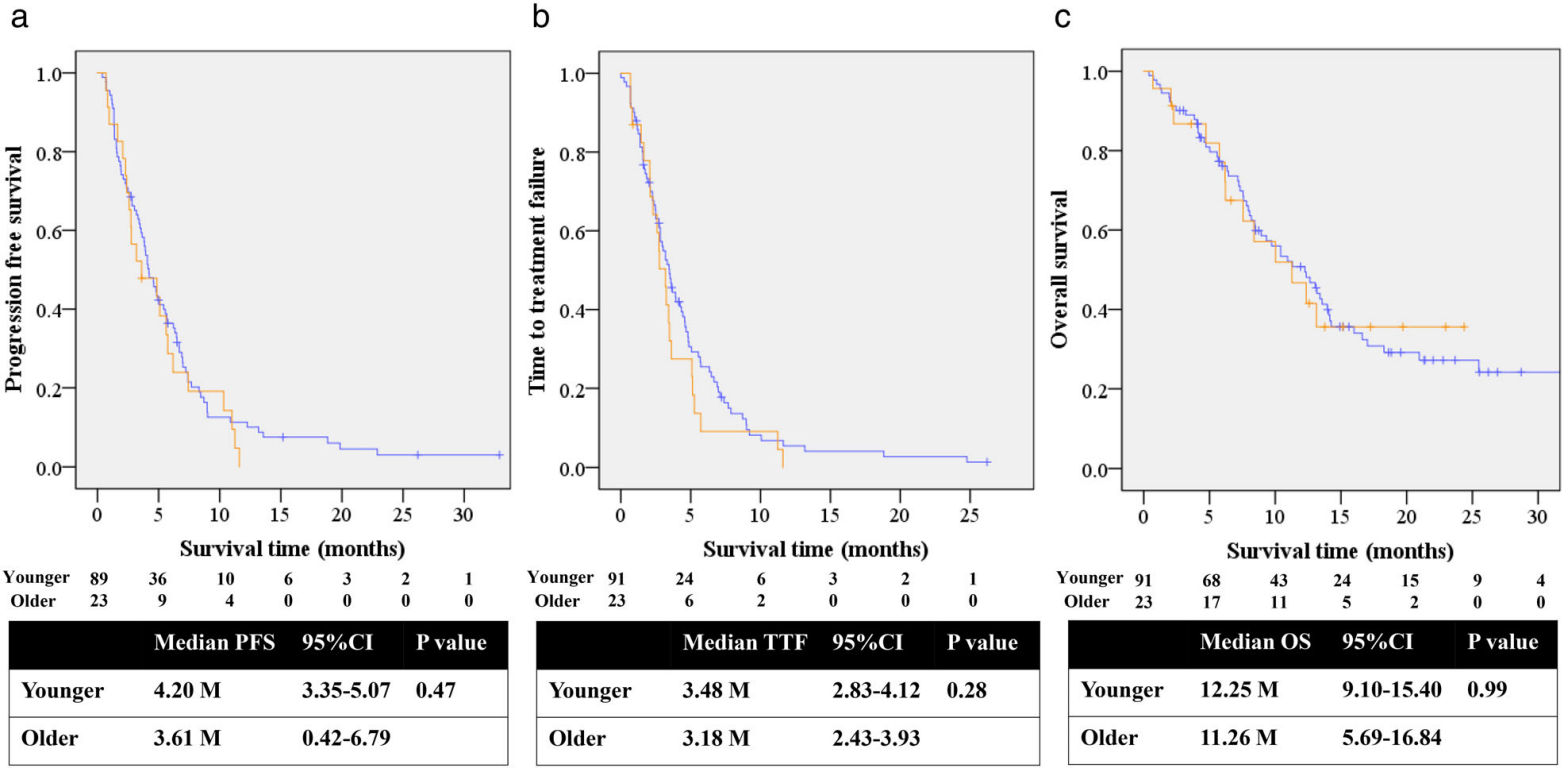
Survival curves by age. (**a**) Progression‐free survival (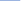) Younger (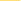) Older. (**b**) Time to treatment failure (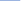) Younger (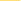) Older. (**c**) Overall survival (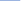) Younger (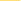) Older.

At data cutoff (April 2019), the median follow‐up was 9.1 months. One older patient (4.3%) and eight younger patients (8.7%) received continuous RAM+DOC treatment. The median number of cycles of RAM+DOC was four for each group. The median PFS, TTF, and OS was 3.6 months (95% CI: 0.4–6.7), 3.1 months (95% CI: 2.4–3.9) and 11.2 months (95% CI: 5.6–16.8) in older patients, and 4.2 (95% CI: 3.3–5.0), 3.4 (95% CI: 3.3–5.0) and 12.2 (95% CI: 9.1–15.4) in younger patients, respectively. Survival curves for each group nearly overlapped, especially for PFS and OS (Fig 1). Although all patients were assessed for therapeutic response, 12 patients were assessed nonevaluable (NE) due to the lack of assessable images in clinical practice. ORR and DCR were 30.4% (95% CI: 13.2–52.9%) and 56.5% (95% CI: 34.5–76.8%) in the older group, and 35.2% (95% CI: 25.4–45.9%) and 61.5% (95% CI: 50.8–71.6%) for the younger group, respectively (Table 2).

**Table 2:** Overall response by age

	Older group (*N* = 23)	Younger group (*N* = 91)
CR	0 (0%)	2 (2.2%)
PR	7 (30.4%)	30 (33.0%)
SD	6 (26.1%)	24 (26.3%)
PD	5 (20.8%)	25 (27.5%)
NE	2 (8.3%)	10 (11.0%)
ORR (95%CI)	30.4% (13.2–52.9)	35.2% (25.4–52.9)
DCR (95%CI)	56.5% (34.5–76.8)	61.5% (50.8–71.6)

CR, complete response; PR, partial response; SD, stable disease; PD, progressive disease; NE, not evaluated; ORR, overall response rate; DCR, disease control rate; CI, confidence interval.

### Use of prophylactic PEG‐G‐CSF and initial dose adjustment

**Figure 2:**
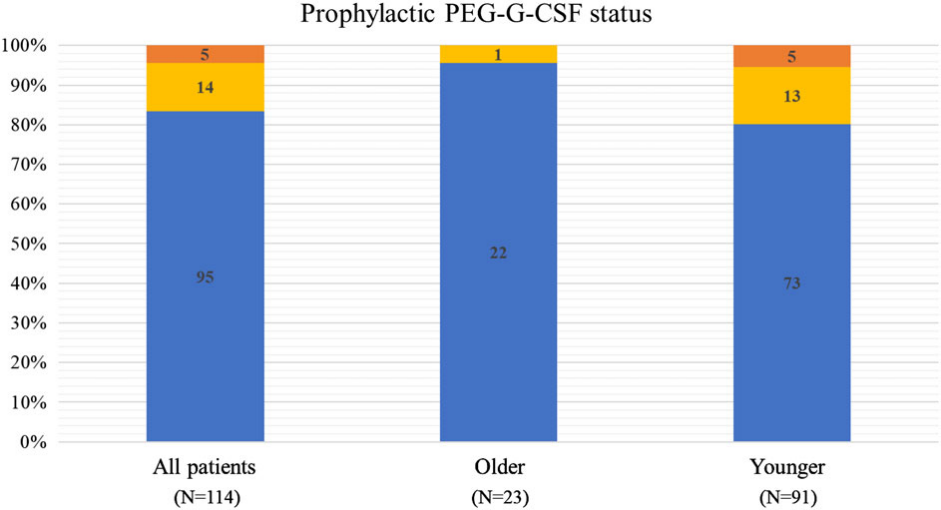
Proportion of prophylactic PEG‐G‐CSF. Prophylactic PEG‐G‐CSF in all patients and age groups. (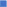) Primary prophylactic PEG‐G‐CSF (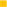) Secondary prophylactic PEG‐G‐CSF (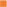) None.

**Figure 3:**
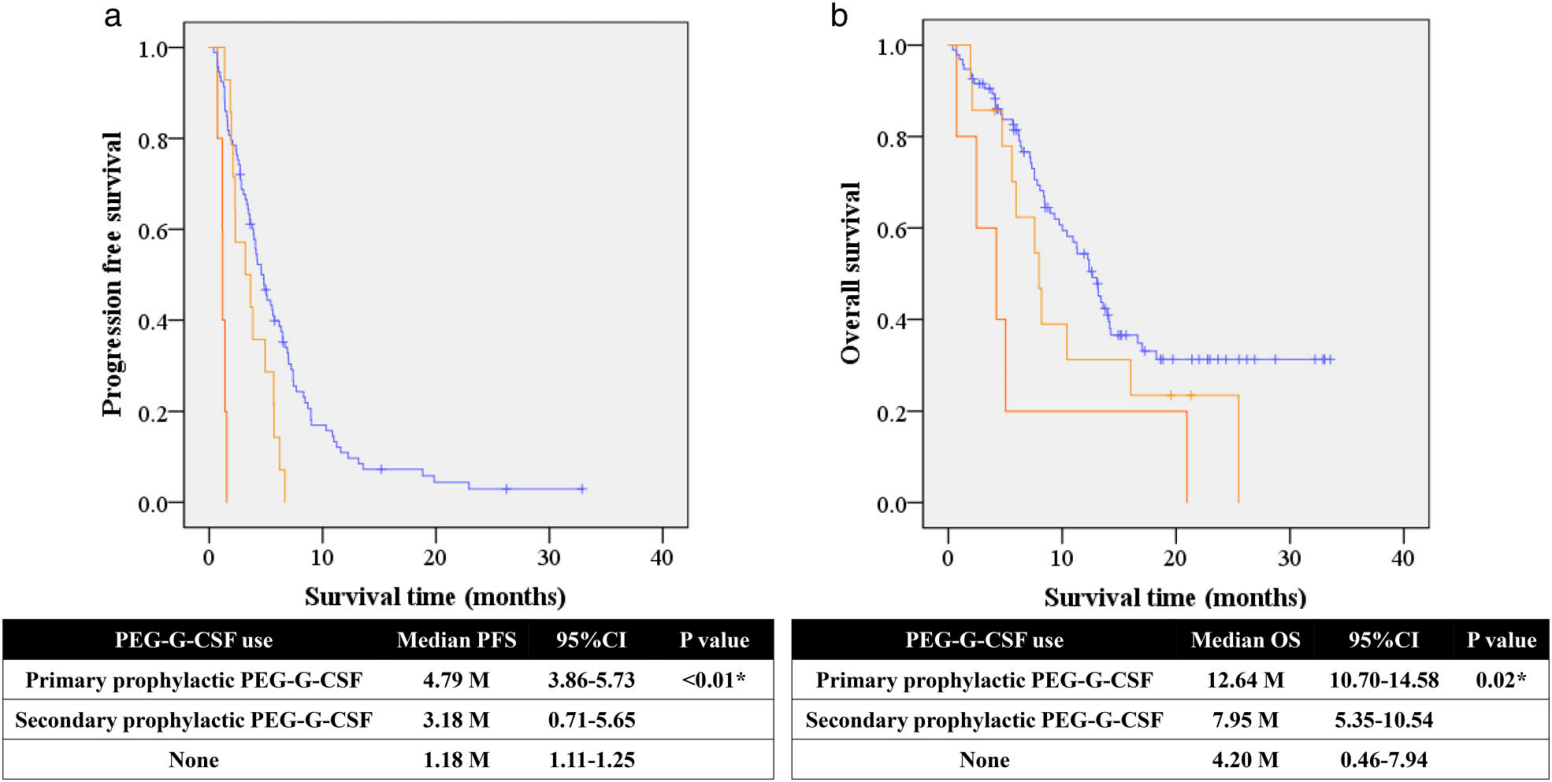
Impact on efficacy of PEG‐G‐CSF. Kaplan‐Meier curves according to prophylactic PEG‐G‐CSF; (**a**) progression‐free survival, (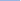) Primary prophylactic PEG‐G‐CSF (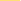) Secondary prophylactic PEG‐G‐CSF (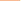) None. (**b**) Overall survival. (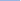) Primary prophylactic PEG‐G‐CSF (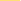) Secondary prophylactic PEG‐G‐CSF (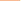) None. *P < 0.05.

In older patients, 22 (95.7%) received primary prophylactic PEG‐G‐CSF, whereas 73 patients (80.2%) received PEG‐G‐CSF in the younger group. One older patient (4.3%) and 13 younger patients (14.3%) received secondary prophylactic PEG‐G‐CSF (Fig 2). The use of prophylactic PEG‐G‐CSF, especially for primary use, was associated with better outcomes for PFS and OS (Fig 3). Multivariate analysis identified the use of primary prophylactic PEG‐G‐CSF as an independent favorable factor for PFS and OS (Tables S2 and S3). Six older patients (26.0%) and five younger patients (5.5%) required a reduction of docetaxel (50 mg/m^2^) at the initial course, while one older (4.3%) and two younger patients (2.1%) required a reduction of ramucirumab (8 mg/kg) at the initial course.

### Safety analysis

In the older group, three patients (13.0%) required a reduction in dosage for regimens after the initial course, whereas, 13 patients (14.3%) received a reduction in the younger group. Four older patients (17.3%) discontinued RAM+DOC due to adverse events which included; one interstitial pneumonia, one anorexia, one diarrhea and one edematous disorder. In the younger group, 14 patients (15.3%) discontinued treatment. Five older patients (21.7%) and 23 younger patients (25.2%) developed Grade ≥ 3 neutropenia. One older patient (4.3%) and nine younger patients (9.8%) required secondary prophylactic PEG‐G‐CSF support after developing febrile neutropenia (FN). In each group, one patient died during RAM+DOC treatment. Key safety data are shown in Table 3.

**Table 3:** Safety profile by age

	Older (*N* = 23)	Younger (*N* = 91)	*P*‐value
Median treatment cycles of RAM (range)	4 (1–8)	4 (1–37)	0.533
Median treatment cycles of DOC (range)	4 (1–8)	4 (1–37)	0.446
Grade ≥ 3 all AE	11 (47.8%)	45 (49.4%)	1
Grade ≥ 3 hematotoxicity	7 (30.4%)	31 (34.0%)	0.809
Grade ≥ 3 nonhematotoxicity	6 (26.0%)	19 (20.8%)	0.582
Grade ≥ 3 neutropenia	5 (21.7%)	23 (25.2%)	1
Febrile neutropenia	1 (4.3%)	9 (9.8%)	0.684
Dose reduction due to AE	3 (13.0%)	13 (14.3%)	1
Discontinuation due to AE	4 (17.3%)	14 (15.3%)	0.758
Treatment‐related death	1 (4.3%)	1 (1.1%)	0.364

AE, adverse event; DOC, docetaxel; RAM, ramucirumab.

## Discussion

This is the first report to investigate the efficacy and safety of RAM+DOC and primary prophylactic PEG‐G‐CSF focused on older patients with advanced NSCLC. In this study, RAM+DOC was efficacious and well tolerated in older patients. RAM+DOC had been considered a high‐risk regimen since the incidence of FN is higher with RAM+DOC (34.2%) than with placebo‐docetaxel (19.8%) in a Japanese phase II study.^9^ However, in our study, it is notable that most patients in the older group received primary prophylactic PEG‐G‐CSF without developing FN. Recent studies report that prophylactic PEG‐G‐CSF not only reduces FN incidences,^12^ but was able to maintain dose intensity and improve the prognosis in adjuvant treatments for breast cancer.^13^ In our study, the use of PEG‐G‐CSF might have influenced better efficacy, especially in primary prophylactic PEG‐G‐CSF, which is consistent with a previous study.^13^ Therefore, primary prophylactic PEG‐G‐CSF is considered an important option for better efficacy and safety, but appropriate dose reduction at the initial course and after the initial course is needed in some cases.

The Japan Lung Cancer Society guidelines do not recommend RAM+DOC for patients aged 75 years and older due to the lack of efficacy and safety data.^10^ In subgroup analysis of age in the REVEL trial,^14^ the hazard ratio model using quintile age groupings adjusted for significant prognostic factors showed OS and PFS hazard ratios favored ramucirumab treatment over the control arm in all age groups. However, this study did not evaluate patients aged 75 and older. Furthermore, in the JVCG trial,^9^ patients aged 75 years and older were rarely enrolled, resulting in a lack of clinical trial data for older patients. In recent retrospective studies which included some patients aged 75 years and older treated with RAM+DOC, there were no instances of FN after receiving prophylactic PEG‐G‐CSF support, whereas FN developed in patients not receiving PEG‐G‐CSF.^15^, ^16^ In consideration of our results, the administration of PEG‐G‐CSF seemed to be essential therapy for RAM+DOC, especially in older patients. Therefore, RAM+DOC with prophylactic PEG‐G‐CSF support may be a useful strategy for patients, regardless of age.

The DRAGON study,^17^ a multicenter, prospective, single‐arm, phase II trial of RAM+DOC with primary prophylactic PEG‐G‐CSF support for chemotherapy‐naive older patients with advanced NSCLC is ongoing in Japan. This study will further shed light on the efficacy and safety of RAM+DOC with primary prophylactic PEG‐G‐CSF support without dose reductions at the initial course.

There were several limitations to this study. First, this was a relatively small retrospective study and further evaluation with larger cohorts is required. Second, this study did not compare the efficacy and safety in older patients receiving docetaxel monotherapy alone; therefore, further comparison studies are needed. Third, the administration of RAM+DOC as an early‐line treatment was most prevalent in older patients, which might have affected the favorable OS in this group. Finally, the standard dose of docetaxel in Japan (60 mg/m^2^) is lower than that of the international standard dose (75 mg/m^2^); therefore, we could not assess the efficacy and safety of the higher dose in this study. However, considering docetaxel at 75 mg/m^2^ was associated with a higher rate of neutropenia in East Asian patients, the lower dose of docetaxel at 60 mg/m^2^ was similar to the rate of neutropenia in the REVEL study for non‐Asian patients.

In conclusion, RAM+DOC is expected to be efficacious and tolerable in older patients with prophylactic PEG‐G‐CSF support. Prophylactic PEG‐G‐CSF support may impact not only safety, but efficacy in patients treated with RAM+DOC.

## Disclosure

Matsusaka Municipal Hospital received research grant funding from Novartis, GlaxoSmithKline, AstraZeneca, Daiichi Sankyo, Bayer, and Boehringer Ingelheim. K. Ito has received speaker fees as honoraria from Eli Lilly Japan, Chugai, AstraZeneca, MSD, Boehringer Ingelheim Japan, Ono, and Pfizer Japan. N. Furuya has received speaker fees as honoraria from Eli Lilly Japan, Chugai, AstraZeneca, Bristol Myers Squibb, Taiho, Boehringer Ingelheim Japan, Ono, and Pfizer Japan. O. Hataji received speaker fees as honoraria from Novartis Pharma, AstraZeneca, and Boehringer Ingelheim Japan. M. Mineshita received honoraria from Eli Lilly Japan, Boehringer Ingelheim Japan, AstraZeneca, Bristol Myers Squibb, MSD, and Novartis Pharma.

## Supporting information


**Table S1A.** Patient characteristics
**Table S1B**. EGFR‐TKI treatment in patients harboring EGFR mutation
**Table S2**. Predictors of PFS analyzed by Cox regression model in all patients
**Table S3**. Predictors of OS analyzed by Cox regression model in all patients.
