# Supplementary material for: The efficacy and safety of ramucirumab plus docetaxel in older patients with advanced non‐small cell lung cancer
Source: Thorac Cancer. 2020 Apr 14;11(6):1559–65. doi: 10.1111/1759-7714.13429 (PMC7262941; doi:10.1111/1759-7714.13429)
Supplement: Supplementary file 1 — Table S1A. Patient characteristics Table S1B. EGFR‐TKI treatment in patients harboring EGFR mutation Table S2. Predictors of PFS analyzed by Cox regression model in all patients Table S3. Predictors of OS analyzed by Cox regression model in all patients. [file TCA-11-1559-s001.docx]

**Supplemental data**

**Supplemental Table 1A**

**Patient characteristics**

|  | Older group (≥75) | Younger group (<75) | P-value |
| --- | --- | --- | --- |
|  |  |  |  |
|  | N=23 | N=91 |  |
| Median age (range) | 77 (75-86) | 68 (40-74) | <0.001* |
| Sex |  |  |  |
| Male | 12 (52.2%) | 65 (71.4%) | 0.087 |
| Female | 11 (47.8%) | 26 (28.6%) |  |
| Smoking history |  |  |  |
| Never | 12 (52.2%) | 19 (20.9%) | 0.007* |
| Former/Current | 11 (47.8%) | 72 (79.1%) |  |
| Histology |  |  |  |
| Non-squamous cell | 14 (60.9%) | 73 (80.2%) | 0.06 |
| Squamous cell | 9 (39.1%) | 18 (19.8%) |  |
| ECOG PS |  |  |  |
| 0 | 6 (26.1％) | 9 (9.8%) | 0.095 |
| 1 | 16 (69.6%) | 72 (79.1%) |  |
| 2 | 1 (4.3%) | 10 (11.0%) |  |
| Clinical stage |  |  |  |
| IIIA-IIIC | 2 (8.7%) | 7 (7.7%) | 0.929 |
| IVA-IVB | 16 (69.6%) | 66 (72.5%) |  |
| Recurrence | 5 (21.7%) | 18 (19.8%) |  |
| No. of prior treatments |  |  |  |
| 0 | 0 (0%) | 4 (4.3%) | 0.058 |
| 1 | 9 (39.1%) | 14 (15.3%) |  |
| 2 | 8 (34.8%) | 34 (37.4%) |  |
| ≥3 | 6 (26.1%) | 39 (42.9%) |  |
| PD-L1 status |  |  |  |
| ≥50% | 1 (4.3) | 15 (16.4) | 0.388 |
| 1-49% | 5 (21.7) | 17 (18.6) |  |
| <1% | 9 (39.1) | 25 (27.4) |  |
| not evaluated | 8 (34.8) | 34 (37.3) |  |
| EGFR status |  |  |  |
| Ex 19 del | 5 (21.7%) | 12 (13.1%) | 0.573 |
| Ex 21 L858R | 1 (4.3%) | 6 (6.5%) |  |
| uncommon mutation | 0 (0%) | 0 (0%) |  |
| not evaluated | 0 (0%) | 2 (2.1%) |  |
| Prior bevacizumab treatment |  |  |  |
| Administered | 13 (56.5%) | 39 (42.9%) | 0.253 |
| None | 10 (43.5%) | 52 (57.1%) |  |
| Prior ICI treatment |  |  |  |
| Administered | 13 (56.5%) | 35 (38.5%) | 0.156 |
| None | 10 (43.5%) | 56 (61.5%) |  |
| Later ICI treatment |  |  |  |
| Administered | 6 (26.1%) | 22 (24.1%) | 1 |
| None | 17 (73.9%) | 69 (75.9%) |  |
| Prophylactic PEG-G-CSF |  |  |  |
| Primary | 22 (95.6%) | 73 (80.2%) | 0.195 |
| Secondary | 1 (4.3%) | 13 (14.2%) |  |
| None | 0 (0%) | 5 (5.4%) |  |

Abbreviations: ECOG PS, Eastern Cooperative Oncology Group performance status; EGFR, epithelial growth factor receptor; ICI, immuno-checkpoint inhibitor; PD-L1, programmed cell death-ligand 1; PEG-G-CSF, pegylated-granulocyte-colony stimulating factor.

*P<0.05

**Supplemental Table 1B**

**EGFR-TKI treatment in patients harboring EGFR mutation**

|  | Older group (≥75) | Younger group (<75) | P-value |
| --- | --- | --- | --- |
|  |  |  |  |
|  | N=6 | N=18 |  |
| Prior EGFR-TKI treatment |  |  |  |
| Administered | 6 (100%) | 18 (100%) | ‐ |
| None | 0 (0%) | 0 (0%) |  |
| Later EGFR-TKI treatment |  |  |  |
| Administered | 2 (33.3%) | 10 (55.5%) | 0.64 |
| None | 4 (66.6%) | 8 (44.4%) |  |

Abbreviations: EGFR, epithelial growth factor receptor; TKI, tyrosine kinase inhibitor.

**Supplemental Table 2**

**Predictors of PFS analyzed by Cox regression model in all patients**

| Variables | Univariate analysis | | Multivariate analysis | |
| --- | --- | --- | --- | --- |
|  | HR (95% CI) | P-value | HR (95% CI) | P-value |
| Age (≥75/<75) | 1.18 (0.73-1.90) | 0.47 |  |  |
| Sex (Female/Male) | 0.75 (0.50-1.13) | 0.17 |  |  |
| Smoking history  (Ever/Never) | 1.05 (0.68-1.62) | 0.79 |  |  |
| Histology (Non-Sq/Sq) | 0.71 (0.45-1.13) | 0.15 |  |  |
| Clinical Stage (IV+R/III) | 2.73 (1.10-6.75) | 0.02 | 2.87 (1.14-7.19) | 0.02 |
| EGFR mutation status  (Mutant/Wild) | 0.48 (0.29-0.85) | <0.01 | 0.42 (0.25-0.71) | <0.01 |
| PD-L1 status (≥1%/<1%) | 1.08 (0.66-1.78) | 0.74 |  |  |
| ECOG PS (2/0-1) | 2.13 (1.12-4.05) | 0.01 | 2.02 (1.06-3.86) | 0.03 |
| Treatment line (≤3rd/≥4th) | 0.73 (0.49-1.10) | 0.14 |  |  |
| Prior ICI treatment (+/‐) | 0.68 (0.46-1.01) | 0.05 | 0.57 (0.38-0.86) | <0.01 |
| Prior BEV treatment (+/‐) | 0.98 (0.66-1.44) | 0.91 |  |  |
| Prophylactic PEG-G-CSF  (primary/secondary+none) | 2.50 (1.48-4.22) | <0.01 | 2.41 (1.42-4.11) | <0.01 |

Abbreviations: BEV, bevacizumab; CI, confidence interval; ECOG PS, Eastern Cooperative Oncology Group performance status; EGFR, epithelial growth factor receptor; HR, hazard ratio; ICI, immuno-checkpoint inhibitor; PD-L1, programmed cell death-ligand 1; PEG-G-CSF, pegylated-granulocyte-colony stimulating factor; R, postoperative recurrence; Sq, squamous cell carcinoma.

**Supplemental Table 3**

**Predictors of OS analyzed by Cox regression model in all patients.**

| Variables | Univariate analysis | | Multivariate analysis | |
| --- | --- | --- | --- | --- |
|  | HR (95% CI) | P-value | HR (95% CI) | P-value |
| Age (≥75/<75) | 0.99 (0.54-1.82) | 0.99 |  |  |
| Sex (Female/Male) | 0.88 (0.53-1.46) | 0.63 |  |  |
| Smoking history  (Ever/Never) | 1.06 (0.62-1.82) | 0.81 |  |  |
| Histology (Non-Sq/Sq) | 0.68 (0.38-1.22) | 0.20 |  |  |
| Clinical Stage (IV+R/III) | 1.96 (0.71-5.40) | 0.18 |  |  |
| EGFR mutation status  (Mutant/Wild) | 0.72 (0.40-1.29) | 0.27 |  |  |
| PD-L1 status (≥1%/<1%) | 1.51 (0.82-2.78) | 0.17 |  |  |
| ECOG PS (2/0-1) | 3.72 (1.86-7.45) | <0.01 | 3.59 (1.79-7.21) | <0.01 |
| Treatment line (≤3rd/≥4th) | 1.07 (0.67-1.72) | 0.75 |  |  |
| Prior ICI treatment (+/‐) | 1.22 (0.75-1.98) | 0.4 |  |  |
| Prior BEV treatment (+/‐) | 1.21 (0.75-1.94) | 0.41 |  |  |
| Prophylactic PEG-G-CSF  (primary/secondary+none) | 1.86 (1.06-3.26) | 0.02 | 1.78 (1.02-3.13) | 0.04 |

Abbreviations: BEV, bevacizumab; CI, confidence interval; ECOG PS, Eastern Cooperative Oncology Group performance status; EGFR, epithelial growth factor receptor; HR, hazard ratio; ICI, immuno-checkpoint inhibitor; PD-L1, programmed cell death-ligand 1; PEG-G-CSF, pegylated-granulocyte-colony stimulating factor; R, postoperative recurrence; Sq, squamous cell carcinoma.
